# Wavelet Domain Radiofrequency Pulse Design Applied to Magnetic Resonance Imaging

**DOI:** 10.1371/journal.pone.0141151

**Published:** 2015-10-30

**Authors:** Andrew M. Huettner, Nikolai J. Mickevicius, Ali Ersoz, Kevin M. Koch, L. Tugan Muftuler, Andrew S. Nencka

**Affiliations:** 1 Department of Biophysics, The Medical College of Wisconsin, Milwaukee, Wisconsin, United States of America; 2 Department of Radiation Oncology, The Medical College of Wisconsin, Milwaukee, Wisconsin, United States of America; 3 Department of Neurosurgery, The Medical College of Wisconsin, Milwaukee, Wisconsin, United States of America; 4 Department of Radiology, The Medical College of Wisconsin, Milwaukee, Wisconsin, United States of America; Universitat de Valencia, SPAIN

## Abstract

A new method for designing radiofrequency (RF) pulses with numerical optimization in the wavelet domain is presented. Numerical optimization may yield solutions that might otherwise have not been discovered with analytic techniques alone. Further, processing in the wavelet domain reduces the number of unknowns through compression properties inherent in wavelet transforms, providing a more tractable optimization problem. This algorithm is demonstrated with simultaneous multi-slice (SMS) spin echo refocusing pulses because reduced peak RF power is necessary for SMS diffusion imaging with high acceleration factors. An iterative, nonlinear, constrained numerical minimization algorithm was developed to generate an optimized RF pulse waveform. Wavelet domain coefficients were modulated while iteratively running a Bloch equation simulator to generate the intermediate slice profile of the net magnetization. The algorithm minimizes the L2-norm of the slice profile with additional terms to penalize rejection band ripple and maximize the net transverse magnetization across each slice. Simulations and human brain imaging were used to demonstrate a new RF pulse design that yields an optimized slice profile and reduced peak energy deposition when applied to a multiband single-shot echo planar diffusion acquisition. This method may be used to optimize factors such as magnitude and phase spectral profiles and peak RF pulse power for multiband simultaneous multi-slice (SMS) acquisitions. Wavelet-based RF pulse optimization provides a useful design method to achieve a pulse waveform with beneficial amplitude reduction while preserving appropriate magnetization response for magnetic resonance imaging.

## Introduction

Simultaneous multi-slice (SMS) imaging has been implemented for applications such as human brain imaging with echo planar imaging (EPI) and diffusion acquisitions [1–7]. In magnetic resonance imaging pulse sequences, spin echo refocusing pulses can exhibit a high peak B1 power when multiple slices are simultaneously excited or when a very high slice bandwidth is desired. Without applying techniques to reduce peak power, the maximal radiofrequency (RF) pulse amplitude scales linearly with the number of slices simultaneously excited. The peak B1 amplitude required to achieve a 180-degree rotation of the proton spins can be prohibitive.

A multiplexed-EPI technique has been shown to accomplish high levels of image acquisition acceleration for twice-refocused diffusion spectrum imaging by combining dual-band excitation with dual simultaneous image refocusing [4], thereby reducing the peak RF amplitude compared to a conventional four-band refocusing pulse. Alternatively, time-shifted RF pulses offer a peak B1 reduction for high slice acceleration factors including three bands and six bands simultaneously excited [8]. To address the challenge of high RF energy deposition at ultra-high field strengths such as 7.0 Tesla and above, the Power Independent of Number of Slices (PINS) technique offers a solution for SMS imaging where SAR reduction is needed [9]. The MultiPINS pulse design technique builds upon the PINS technique to further reduce peak power and energy deposition for SMS pulses by mixing a PINS pulse with a more traditional multiband pulse [10].

Variable-rate selective excitation (VERSE) is an approach to reduce peak RF amplitude and specific absorption rate (SAR) with time-varying gradients during the RF pulse [11]. VERSE techniques have been implemented in pulse sequences such as a twice-refocused adiabatic spin echo for immunity to inhomogeneity and RF power reduction [12] and utilized for rapid 3D sequences [13]. Recently, VERSE has been paired with Hadamard encoding to design dual-band SMS pulses for improved diffusion weighted imaging in the spine [14].

Methods based on optimal control theory have been developed to optimize RF pulses for high-tip angle excitation, spin echo refocusing, and selective inversion [15,16]. Asymmetric, purely amplitude modulated spin echo pulses and phase modulated spin echo pulses have been reported to reduce peak RF power compared to symmetric sinc-style pulses [17]. Mao et al. applied conjugate gradient descent methods to solve a nonlinear optimal control problem based on the desired spin magnetization profile while neglecting relaxation effects to design high bandwidth (4.7kHz and 8.4kHz) inversion pulses [16]. A method for point-resolved spectroscopy refocusing pulse design implemented an optimal control algorithm to achieve a bandwidth increase from 0.974 kHz to 2.999 kHz while holding peak B1 constant at 23 μT [18]. Recently, optimal control theory has been applied to design SMS excitation pulses with a cost function based on specific absorption rate (SAR) [19].

Methods of RF phase modulation have been developed to increase the RF bandwidth while keeping peak RF amplitude constant to satisfy the peak B1 constraints on the scanner hardware. Common approaches are based on the Shinnar Le-Roux (SLR) algorithm [20] and involve optimization of SLR polynomials [21,22]. Applications include high bandwidth pulses for spectroscopy, saturation and outer-volume suppression. Pulses with a polynomial phase response have been shown to yield a peak B1 as low as 31% of a linear phase pulse when designed with a peak B1 limit of 20 μT for improved outer-volume suppression [21]. Furthermore, zero-flipping, or root inversion, of the SLR A-polynomial through nonlinear optimization was demonstrated to increase the pulse bandwidth from 1.4 kHz to 3.6 kHz while holding peak B1 constant at 24 μT [22] for point resolved spectroscopy. In these applications, spin echo twice-refocusing provides correction of the nonlinear phase. Pulses that impart a nonlinear phase may be used for saturation and outer-volume suppression. However, for spin echo refocusing, such pulses need to be implemented as a twice-refocused sequence or designed as 90–180 pairs for appropriate phase response. Alternatively, single band nonlinear phase pulses with reduced peak power can be modulated into SMS multiband pulses with optimal phase schedules for an overall peak B1 reduction [23].

Root inversion SLR design techniques have been applied to multiband slice multiplexing [24] and to the design of nonlinear phase excitation and refocusing pulse pairs to minimize RF power for multiband SMS imaging by searching for ideal SLR root combinations [25,26]. In these methods, the ideal refocusing pulse has nonlinear phase and therefore phase-paired excitation pulses need to be used in tandem with refocusing pulses to achieve the desired RF amplitude reduction while preserving through-slice phase coherence.

In this work, we demonstrate the application of wavelet decomposition and reconstruction to RF pulse design through nonlinear numerical optimizations. Wavelet basis functions, when applied to an optimization problem, can reduce the number of unknowns through compression while retaining a high degree of design flexibility. The compression provided through wavelet transforms allows the complex optimization of SMS refocusing pulses with many degrees of freedom to become a tractable optimization problem.

## Algorithms

Discrete wavelet decomposition and reconstruction transformations allow conversion between the time domain and wavelet domain with exact reconstructions. The wavelet domain provides the benefit of selecting a limited number of wavelet coefficients to be used by a nonlinear optimization algorithm. In the absence of wavelet transforms, the parameter space of a numerical solution for an “optimal” RF pulse for a given slice profile is prohibitively large. For a specified number of RF amplitude resolution points M and time domain points N, the number of possible solutions is M^N^. In the extreme case, a digitized 3.2 ms RF pulse on modern MRI equipment offers over 32700^3200^ possible solutions for maximal amplitude resolution. Compression of the parameter space to a more reasonable size is necessary to generate robust numerical solutions in appropriate time frames. In this work, we describe the compression of RF pulses through the wavelet transform with modulation of a reduced set of wavelet coefficients followed by the inverse wavelet transform, Bloch simulation, and cost function evaluation. In this section we describe the wavelet compression in further detail.

### Wavelet Prototypes and Compression

Wavelet basis functions provide compression to improve the conditioning of the cost function minimization. In practice, a wide range of prototype wavelets may be utilized. Specific wavelet prototypes that provide a high level of compression for SMS pulses include the Discrete Meyer wavelets, Symlets (orders 5–9), and Daubechies wavelets (orders 7–9). The spectrum, after Fourier Transform, for each of these three prototype wavelets exhibits a dual-band profile. These prototype wavelets may be utilized for compression of SMS pulses with more than two pass bands. Yet, the highest level of compression is achieved when the prototype wavelet resembles the desired SMS pulse considering the number of pass bands.

**Fig 1:**
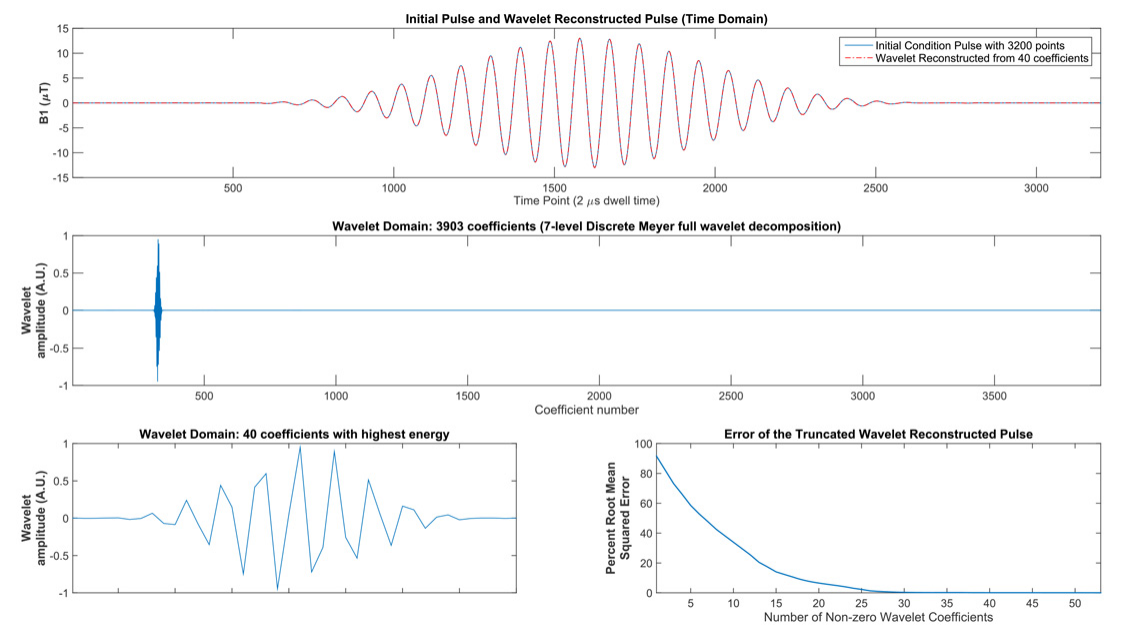
Wavelet compression of a time domain pulse. A 2-band SMS purely amplitude modulated pulse is shown with the corresponding wavelet decomposition. This pulse was selected as the initial condition for the wavelet optimization. The top panel shows the initial condition pulse (blue trace) plotted against the pulse (dashed-dotted red trace) that was wavelet reconstructed exclusively from the 40 wavelet coefficients shown in the bottom panel. The middle panel provides the full wavelet decomposition (using Discrete Meyer wavelet prototypes) exhibiting the sparseness of the wavelet domain. The lower left panel shows the 40 highest energy wavelet coefficients (above the energy threshold of 0.001). The lower right panel plots the root mean squared error (RSME) percentage of the initial condition pulse compared to the wavelet reconstructed pulse after truncation in the wavelet domain to a limited number of coefficients and zero-filling the remainder of the wavelet domain. In this figure, 3200 time domain points were compressed to 40 wavelet domain coefficients representing 98.7% compression of the original time domain pulse.

For wavelet optimization of purely amplitude modulated dual-band pulses, the level of compression is exceptionally high for Discrete Meyer prototype wavelets, with as few as 40 coefficients required to provide a high fidelity wavelet reconstruction for generation of a time domain pulse with 3200 points. The sparseness of the wavelet domain is displayed in Fig 1 for a dual-band initial condition pulse with 600 Hz pass bands and 10.8 kHz of spectral distance between centers of the two bands. The full wavelet decomposition of the 3200-point time domain pulse contains 3903 wavelet coefficients for the 7-level Discrete Meyer decomposition. The wavelet domain is over 98% sparse as shown in Fig 1. The pulse that was reconstructed from only 40 coefficients is plotted on the same axes as the original pulse in Fig 1 (top panel). The trace of the wavelet-reconstructed waveform matches the original pulse trace very well, with no discernible difference in the plot. Reconstruction of 3200 time domain points from 40 wavelet coefficients with zero filling of the remaining 3863 wavelet coefficients yields a nearly exact reconstruction of the original pulse. As shown by the root mean squared error (RMSE) plot in Fig 1, the percent RMSE approaches very low values at 30 non-zero wavelet coefficients and higher. The RMSE is computed according to the equation:

$$\frac{\sqrt{\text{mean}{\left({\text{RF}}_{0}-{\text{RF}}_{\text{w}}\right)}^{2}}}{\sqrt{\text{mean}{\left({\text{RF}}_{0}\right)}^{2}}}$$ (1)

where RF_0_ is the original time domain pulse and RF_w_ is the wavelet reconstructed pulse after truncation to a limited number of wavelet domain coefficients. At 20, 30 and 40 wavelet coefficients, the percent RMSE equals 6.56%, 0.33% and 0.13%, respectively.

### Optimization Algorithm

**Fig 2:**
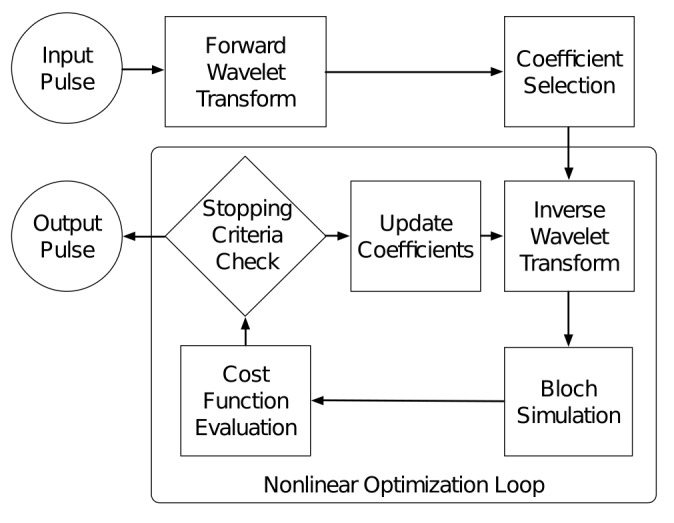
Diagram of the pulse optimization workflow. Wavelet decomposition is performed on the initial condition (input) pulse. Truncation in the wavelet domain selects the desired number of coefficients based on an energy threshold and the remaining coefficients are set to zero. The nonlinear optimization loop iteratively evaluates the cost function until the stopping criteria are satisfied. The final coefficients are wavelet reconstructed to obtain the ideal time domain pulse.

The compression properties of the Discrete Meyer wavelets shown in Fig 1 can be applied to a radiofrequency (RF) pulse design problem for magnetic resonance spin echo refocusing pulses. Based on wavelet transform functions, a nonlinear, constrained numerical optimization was written in MATLAB (R2014B, Wavelet and Optimization Toolboxes, The Mathworks, Inc.). This software uses the MATLAB wavelet decomposition and wavelet reconstruction functions, the MATLAB nonlinear constrained algorithm *fmincon*, and an open source Bloch equation simulator [27]. The optimization algorithm follows the workflow shown in Fig 2. An initial pulse undergoes a wavelet transform wherein a subset of non-zero wavelet coefficients is selected. Through an inverse wavelet transform, this subset of coefficients generates a radio frequency pulse in the time domain. The resulting radio frequency pulse is run through a Bloch simulation and the simulated excitation profile is evaluated with a cost function. Iteratively, the amplitudes of the coefficients from the truncated wavelet decomposition are adjusted throughout the nonlinear optimization algorithm.

## Methods

The nonlinear iterative minimization described in this work was used to generate a wavelet-optimized refocusing pulse for a spin echo diffusion EPI pulse sequence. Simulations were completed based on Bloch simulation for evaluation of pulse performance. Next, the wavelet-optimized pulse, along with existing Fourier and SLR pulses, was implemented in a diffusion pulse sequence to obtain human brain images at 7.0 Tesla.

### Optimization Design and Implementation

The cost function is based on the slice profile from Bloch simulation compared to the ideal slice profile, with additional terms. The L2-norm is computed by taking the sum of the squared difference of the simulated slice profile compared to the ideal slice profile. The slice profile is based on the M_x_, M_y_ and M_z_ components after a 72.8 ms Bloch simulation that includes the full sequence of excitation and refocusing pulses with simulated gradients. The cost function equation is shown below.

$$\text{X}=\sum_{\text{f}}{\left({\text{M}}_{\text{f}}-{\text{M}}_{\text{f},\text{ideal}}\right)}^{2}+\lambda_{1}\sum_{\text{f}}{\left(\Theta_{\text{f}}-\Theta_{\text{f},\text{ideal}}\right)}^{2}+\lambda_{2}\sum_{\text{b}}{\left(\eta_{\text{b}}-\zeta_{\text{b}}\right)}^{2}$$ (2)

**Fig 3:**
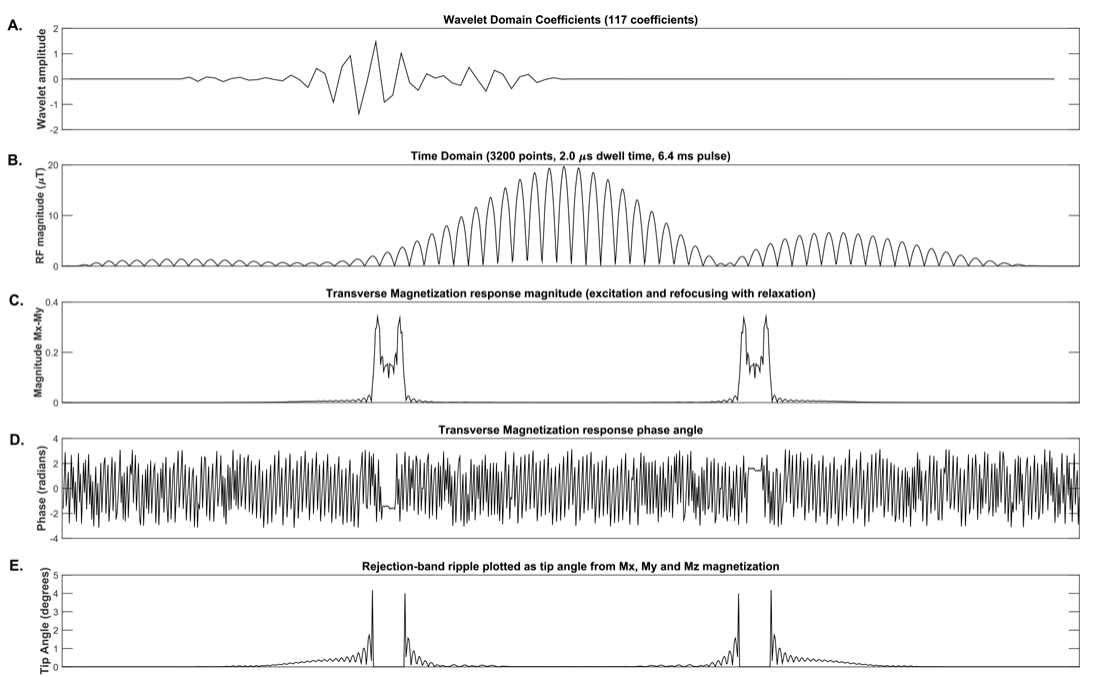
Graphical display of wavelet coefficients and cost function terms for a 2-band pulse. Panel (A) at the top shows the 117 wavelet domain coefficients whose amplitudes were optimized. The amplitude modulated time domain pulse with 3200 points shown in panel (B) was generated from the wavelet coefficients displayed in the top panel. Panel (C) shows the transverse magnetization profile at the echo time after Bloch Simulation of excitation and refocusing pulses. Panel (D) displays the phase of the transverse magnetization at the echo time. Panel (E) shows the tip angle for solely the rejection band that was dynamically computed from the net magnetization vector. The lower three plots represent the spin magnetization state from Bloch simulation for a single spin isochromat on resonance at each spatial frequency point.

In the first term, M_f_ is the magnitude of the transverse magnetization for each spectral offset frequency, computed as $\sqrt{{\text{M}}_{\text{x}}{}^{2}+{\text{M}}_{\text{y}}{}^{2}}$. Magnetization vectors M_x_ and M_y_ come from Bloch simulation after wavelet reconstruction using the wavelet coefficients being optimized. The variable M_f,ideal_ represents the ideal target profile with pass bands equal to the desired value of the magnetization and zeros outside the pass bands. The first term is shown graphically in Fig 3 (panel C).

The second term of the equation penalizes the rejection band ripple based on Θ_f_ which is the tip angle in degrees for each location in the spectral slice profile outside of the slice pass bands. A regularization factor for this term is denoted by λ_1_. The value of λ_1_ was set to 0.01, determined empirically to adjust for the scaling difference between M_f_ and Θ_f_. The tip angle was computed using the magnetization vectors from Bloch Simulation according to the equation:

$$\Theta_{\text{f}}={\text{cos}}^{-1}\frac{{\text{M}}_{\text{z}}}{\left|{\text{M}}_{\text{xyz}}\right|}$$ (3)

In this equation, M_z_ is the z-component of the magnetization and M_xyz_ represents the net magnetization vector with magnitude computed as $|{\text{M}}_{\text{xyz}}|=\sqrt{{\text{M}}_{\text{x}}{}^{2}+{\text{M}}_{\text{y}}{}^{2}+{\text{M}}_{\text{z}}{}^{2}}$. The output from this term, as applied to the cost function, is plotted in Fig 3 (panel E). Computing the tip angle with this equation was chosen to provide scaling based on tip angle, rather than longitudinal magnetization alone. This scaling has the benefit of improved control to minimize stop band ripple as well as sensitivity to undesired inversion bands (tip angles near 180 degrees), compared to evaluating solely the longitudinal or transverse component of the magnetization.

The third term in the cost function maximizes the phase coherence of the transverse magnetization by computing the transverse vector sum across each slice pass band compared to the ideal vector sum value. The difference between the actual and ideal vector sums is squared and summed for each band. This term is multiplied by an additional regularization parameter λ_2_. The parameter λ_2_ is computed based on the Bloch simulation response from the initial condition input pulse and was determined empirically to equal 827.45 for this optimization. The simulated vector sum of the transverse magnetization and the ideal vector sum value for each pass band, b, are denoted by scalars η_b_ and ζ_b_, respectively. The scalar, η_b_, is computed from the transverse magnetization components at frequencies, f, contained in each pass band:

$$\eta_{\text{b}}=\left|\sum_{\text{f}\in \text{b}}{\text{M}}_{{\text{x}}_{\text{f}}}+\text{i}{\text{M}}_{{\text{y}}_{\text{f}}}\right|$$ (4)

This third term comes from the M_x_ and M_y_ magnitude and phase vectors plotted in Fig 3 (panels C and D). The three terms described above are added together to provide the intermediate cost function value that is returned to the iterative minimization algorithm (*fmincon* in MATLAB).

The cost function terms for the optimization algorithm are shown graphically in Fig 3. The initial condition pulse was based on the Fourier transform (small tip angle) approximation of the ideal slice profile. This Fourier dual-band pulse was utilized to determine the significant wavelet coefficients by performing a wavelet decomposition followed by selection of those coefficients with wavelet domain amplitude greater than the desired threshold of 7.0x10^-5^ (for reference, the coefficient with highest energy has wavelet amplitude of 9.516x10^-1^). Based on this threshold, 117 wavelet coefficients were selected using Discrete Meyer prototype wavelets.

The optimization was designed to incorporate Bloch simulation of a spin echo sequence including excitation and refocusing pulses paired to constant slice-select gradients with gradient crushers surrounding the refocusing pulse. Excitation pulses were generated via previously published methods [5] and held constant for the optimization. For the wavelet pulse optimization shown in Fig 3, one spin isochromat at each spatial offset frequency location was simulated over a spectral range of 30 kHz with 30 Hz steps (corresponding to a spatial range of 200 mm with 0.2 mm steps). Bloch simulation included spin relaxation effects using the spin-lattice relaxation time (T_1_) of 1300ms and a spin-spin relaxation time (T_2_) of 38ms. The echo time was 72.8ms for the full spin echo Bloch simulation including a dual-band excitation pulse followed by the dual-band wavelet-optimized refocusing pulse shown in Fig 3, panel B.

In MATLAB (The Mathworks, Natick, MA), the function *“fmincon”* was utilized for constrained nonlinear optimization. Three different minimization algorithms in MATLAB (optimization toolbox) available with the *fmincon* function were evaluated for performance with RF pulse optimization: the interior-point algorithm, the active-set algorithm and the sequential quadratic programming (SQP) algorithm. The SQP algorithm was chosen for wavelet optimization based on its characteristics of the highest speed and quickest convergence while yielding a result almost identical to the other two algorithms. The default MATLAB convergence criteria was used for the algorithm which stopped at step length = 4.748x10^-3^, Euclidean norm of step = 2.501 x10^-6^ and first-order optimality = 1.793 x10^-3^. For a purely amplitude modulated pulse such as the linear-phase dual-band pulses presented in this work, only the real (or imaginary) component is applicable because the other equals zero. A constant slice-select gradient was used and the pulse width remained constant at 6.4ms for the duration of the optimization.

For control of peak B1 for the pulse optimization, the minimization cost function accepts as input a maximum peak B1 value. During the iterative minimization process, it may occur that the algorithm chooses wavelet coefficient amplitudes that, after wavelet reconstruction, would exceed the time domain peak B1 constraint. In this case, the cost function dynamically rescales the time domain pulse amplitudes (immediately after wavelet reconstruction) to satisfy the desired peak B1 constraint. Additionally, this cost function peak B1 variable may be used to design a wavelet-optimized pulse that has a lower peak B1 amplitude compared to the initial condition pulse.

### Simulations

For comparison to the wavelet optimized pulse, a multiband SMS pulse was designed according to the Shinnar-Le Roux (SLR) algorithm with MATLAB code developed in-house. This code utilizes open source SLR routines [28] and is based on work by Cunningham and Wood for generation of SMS multiband SLR pulses [29]. For a direct simulated comparison of the magnetization response, Bloch Simulation was run separately on the SLR, Fourier and wavelet-optimized dual band pulses with 100 normally distributed spin isochromats spanning an off-resonance frequency range of 51.25 Hz with a standard deviation of 12.44 Hz at each spatial location. This Bloch simulation incorporated excitation and refocusing pulses with gradient crushers surrounding the refocusing pulse to yield a crushed spin echo response. The time step (dwell time) was 2 microseconds for both the RF and gradient waveform arrays. To achieve a slice thickness of 4.0 mm with these pulses, the simulated slice-select gradient was 7.3 mT/m (1.25 kHz pass bands) for the excitation pulses and 3.5 mT/m (600 Hz pass bands) for the refocusing pulses.

### Ethics Statement

Human brain images were acquired on a GE Healthcare Discovery MR950 7.0T scanner. A healthy human subject was scanned after written informed consent was obtained under a protocol approved by the Human Research Protection Institutional Review Board (IRB) at the Medical College of Wisconsin.

### Imaging Experiment Design

To evaluate the new dual-band RF pulses, four separate human brain scans were acquired consecutively with the same slice locations, echo time (TE) and repetition time (TR). These scans included a single-band acquisition followed by dual-band acquisitions with Fourier, SLR, and wavelet-optimized refocusing pulses. For these three refocusing pulses, the peak B1 amplitudes were as follows: 13.0 μT for the Fourier dual-band refocusing pulse, 21.7 μT for the SLR dual-band refocusing pulse, and 19.7 μT for the wavelet-optimized dual-band refocusing pulse. The bandwidth of the RF pulse pass bands was chosen as 600 Hz in order to design a SLR dual-band refocusing pulse to easily meet peak B1 constraints of the scanner without implementing advanced SLR design techniques for peak B1 reduction [22,25,26,30]. The RF pulse width was 6.4ms for each of the dual-band refocusing pulses as well as the dual-band excitation pulse.

The standard EPI diffusion pulse sequence was modified to incorporate custom dual-band excitation and refocusing pulses. The diffusion tensor imaging (DTI) pulse sequence is a single-shot EPI, single-refocused spin echo sequence. A Nova Medical 2-channel head-only transmit coil, operating in quadrature transmit mode, was paired with a Nova Medical 32-ch receive brain array. Eighteen sagittal aliased slices were acquired in each TR period to yield 36 separated slices after reconstruction for whole brain coverage. A single band scan, using the standard diffusion (DTI) pulse sequence on the scanner, was acquired for comparison with our customized dual-band pulse sequence.

Scan parameters included a 4.0 sec TR, 73.2 ms TE, 4 mm isotropic voxels, 25.6 cm field of view, 4 mm slice thickness, and a 64x64 acquisition matrix with full k-space readout. A low resolution, full k-space acquisition was selected to minimize potential reconstruction-related confounds associated with in-plane acceleration and partial k-space while minimizing echo time. Two reference diffusion images for each slice were acquired with no diffusion gradients (b = 0 s/mm^2^) followed by 30 DTI directions at b = 1000 s/mm^2^. At a TR of 4.0 sec, 36 slices were acquired in one TR for the single-band acquisition and 18 slices were acquired for the dual-band acquisition. Although the TR period could be shortened for the dual-band acquisitions to provide acceleration, the TR period was held constant for all four scans to provide a direct comparison without changes in scan parameters. Images were reconstructed from the raw data file using the GE Healthcare Orchestra reconstruction software development kit in MATLAB. To separate the dual-band slices, a sensitivity encoding (SENSE) [31] reconstruction of our own design was implemented within the Orchestra reconstruction pipeline to obtain the acceleration factor of two in the slice direction. A separate calibration scan was used for SENSE calibration.

## Results

**Fig 4:**
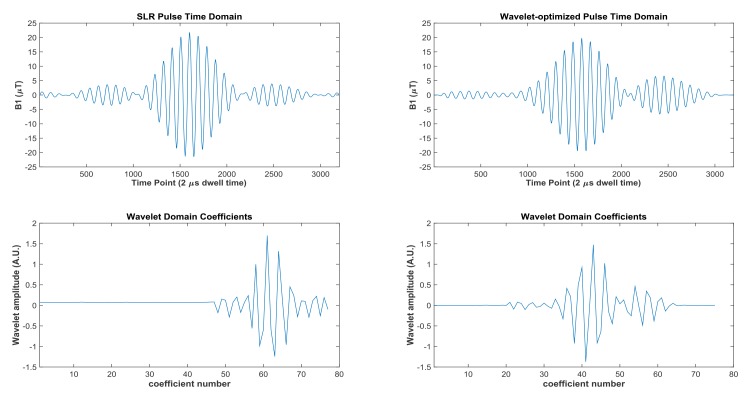
Wavelet coefficients for Shinnar-LeRoux (SLR) and wavelet time domain pulses. A 2-band SMS Shinnar-LeRoux (SLR) purely amplitude modulated pulse is shown (peak B1 of 21.69 μT) along with the corresponding wavelet decomposition on the left column. The right column shows the amplitude modulated wavelet optimized 2-band SMS pulse yielding the same slice bands as the SLR pulse. The wavelet optimization achieved a peak B1 of 19.67 μT (reduction of 10.7% from the comparable SLR pulse). The lower row shows the wavelet coefficients containing the highest energy. In this example, 3200 time domain points were compressed to 75 wavelet domain coefficients.

A wavelet-optimized dual-band pulse was designed by starting with the Fourier pulse as the initial condition for the minimization algorithm. After convergence was reached, the wavelet-optimized pulse yields a spectral slice profile response nearly identical to the SLR profile. This similarity is in spite of the fact that the SLR pulse was kept entirely separate from the wavelet optimization algorithm and only the Fourier pulse was used as an input to the optimization. In this optimization, the peak B1 constraint for the wavelet optimization was set at 21.69 μT to match the peak B1 amplitude of the comparable SLR pulse. For the wavelet-optimized pulse shown in Fig 4, the algorithm converged at a lower peak B1 of 19.67 μT.

### Simulation Results

The wavelet-optimized pulse is compared in Fig 4 to an SLR pulse that was designed based on the methods described by Cunningham and Wood [29]. The wavelet pulse provides the advantages over the full-power SLR pulse of a slightly lower peak B1 and lower ripple in between the pass bands, as well as lower SAR. Worth noting, the rejection band ripple in the SLR pulse could be further reduced if a higher peak B1 amplitude is deemed tolerable. The integral of B1 squared of the wavelet pulse is 198.0 relative to 220.9 (μT^2^ ms) for the SLR pulse. The wavelet and SLR pulses have the advantages over the Fourier pulse by achieving a slice profile with steeper and shorter transition bands and a higher pass-band vector sum net signal compared to the Fourier pulse, based on Bloch Simulation. Although the Fourier pulse exhibits the lowest peak B1 value (listed in Table 1), this comes at the expense of poor spectral performance. Table 1 provides a quantitative comparison of the properties of the Fourier, SLR and wavelet refocusing pulses. Compared to the SLR pulse, the wavelet-optimized pulse achieves a 10.7% reduction in peak B1 and a 9.6% reduction in the integral of B1 squared while retaining a comparable transverse magnetization vector sum value for each pass band (Table 1).

**Table 1:** Comparison of three dual-band pulses.

	Fourier Pulse	SLR Pulse	Wavelet Pulse
Peak B1 (μT)	13.00	21.69	19.67
Integral of B1 squared (μT^2^•ms)	112.0	220.9	198.0
Real-time SAR during human scan (W/Kg)	0.7	1.1	1.0
Relative vector sum across a slice pass-band from Bloch simulation (band 1)	0.352	0.502	0.505
Relative vector sum across a slice pass-band from Bloch simulation (band 2)	0.353	0.502	0.506
Rejection band ripple at the center carrier frequency	0.00004%	0.48%	0.026%

Pulse properties are shown for the Fourier, SLR and wavelet-optimized refocusing pulses implemented in a spin echo diffusion EPI pulse sequence.

**Fig 5:**
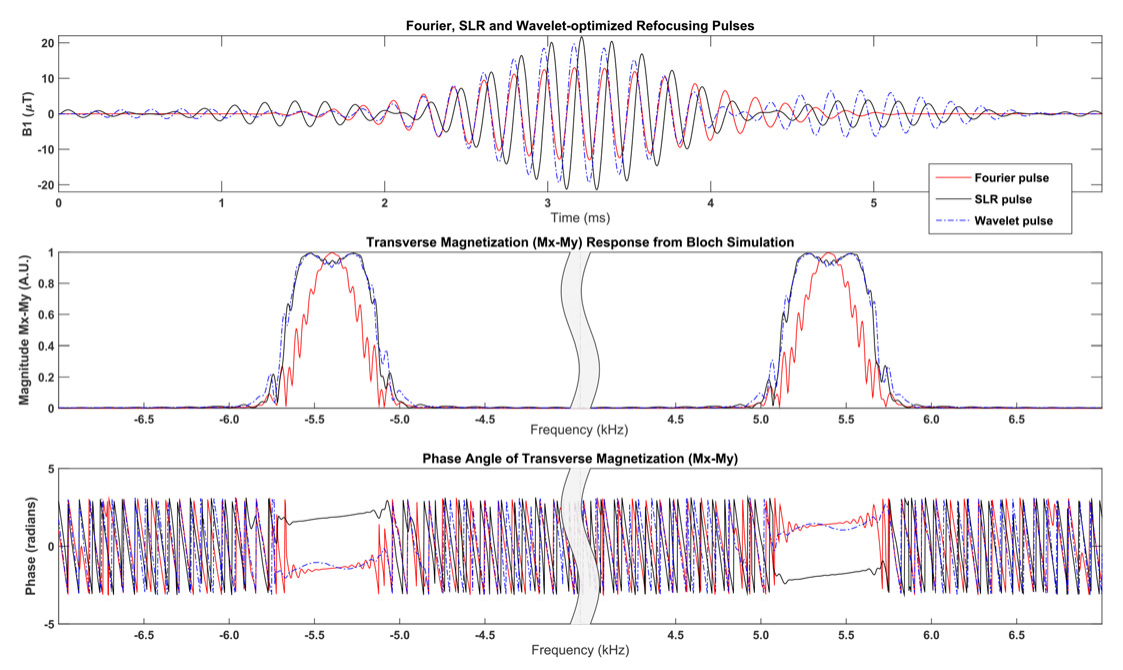
Bloch Simulation comparison of three dual-band refocusing pulses. For the three pulses shown (Fourier pulse, SLR pulse, or Wavelet pulse), Bloch simulation comprised a dual-band excitation pulse followed by one of the three dual-band refocusing pulses plotted in the top panel with 50 spin isochromats and an echo time of 72 ms to simulate a crushed spin echo response. The second panel compares the transverse magnetization magnitude zoomed to each of the two pass bands. The SLR and wavelet pulse exhibit a very similar profile. The bottom panel plots the phase angle in radians of the transverse magnetization for each pulse.

Simulation results were obtained from Bloch simulation to compare the magnetization response for different dual-band refocusing pulses. Fig 5 compares the Fourier, SLR and wavelet-optimized refocusing pulses for a crushed spin echo Bloch simulation with 50 normally distributed isochromats (standard deviation of 12.75 Hz) at each spatial location. The same Fourier excitation pulse was used for all three simulations followed by different refocusing pulses, as shown in the top panel in Fig 5. The excitation pulse has 1.25 kHz pass bands and each of the three refocusing pulses have 600 Hz pass bands. Although the SLR and wavelet-optimized pulses exhibit higher peak B1 amplitude compared to the Fourier pulse, the slice profile for the SLR and wavelet-optimized pulses is a much closer fit to the ideal (rectangular) slice profile. The transition bands are very sharp on the wavelet pulse and the slice profile closely resembles the profile of an SLR pulse as plotted in the middle panel of Fig 5. The phase angle of the transverse magnetization response is plotted in Fig 5 as the bottom panel. Although the phase angle across the pass band appears different for the wavelet pulse (blue dashed trace, Fig 5) compared to the SLR pulse (black trace, Fig 5), the net signal measured by the coil is expected to be the same. The amplitude of phase variation across a pass band for the wavelet pulse response is less than the total change in phase angle (maximum less minimum) across the same pass band. Furthermore, the vector sum of magnetization vectors M_x_ and M_y_ was computed for the pass band and found to be almost identical for the SLR compared to the wavelet pulse, as shown in Table 1.

### Human Imaging Experiments

**Fig 6:**
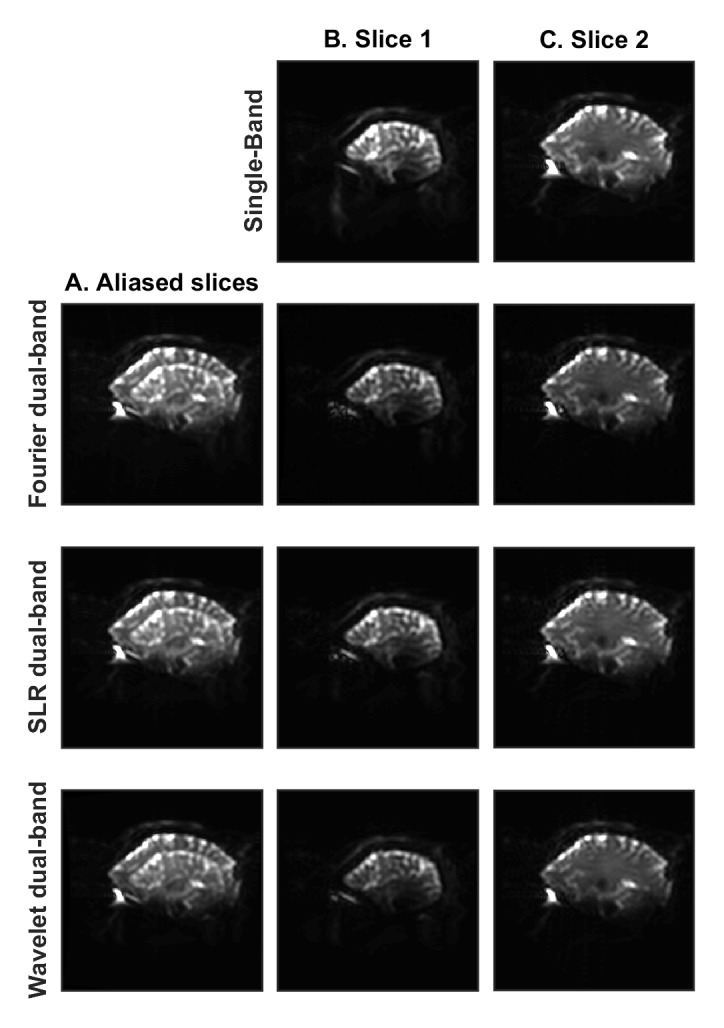
Human Brain Images. Reconstructed sagittal brain images obtained with the standard single-band pulse sequence on the General Electric MR950 7.0T scanner are compared to images obtained with the dual-band Fourier, SLR and wavelet-optimized refocusing pulses. All images are shown with the same window level. Single-band images are shown in the top row with standard reconstruction on the scanner. Column A shows the acquired (aliased) images containing two different brain slices superimposed. The two separated slices, after SENSE reconstruction, are shown in column B and column C for the Fourier, SLR and wavelet-optimized refocusing pulses. Other than differing refocusing pulses, all aspects of the scan sequence remained the same.

**Fig 7:**
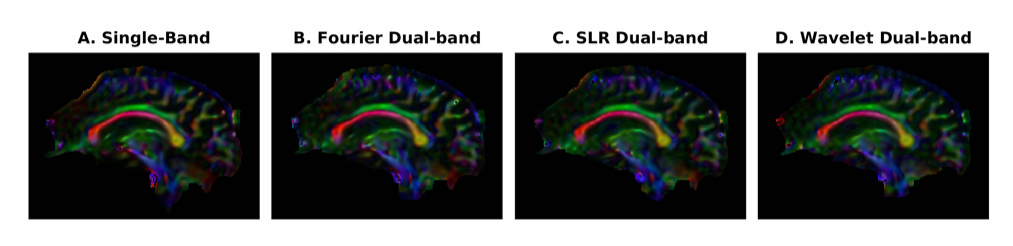
Diffusion (DTI) color-coded fractional anisotropy (FA) maps. The FA maps compare a central sagittal slice for the standard single-band diffusion pulse sequence (panel A) with our SMS sequence using Fourier, SLR and wavelet-optimized refocusing pulses (panels B, C and D). The customized SMS pulse sequence, after reconstruction, yields comparable results the standard sequence (panel A) acquired without slice acceleration. The scan parameters included a 4.0 sec TR, 73.2 ms TE, 30 DTI directions and b = 1000 s/mm^2^. The color-coding scheme denotes the principal diffusion direction as red for left-right, green for anterior-posterior, and blue for superior-inferior. The level of brightness represents the magnitude of the FA value.

Human brain images are shown in Fig 6. Clear separation of the slices was achieved with the 2-band excitation pulses followed by wavelet-optimized refocusing pulses. Diffusion image analysis was completed using the software packages AFNI (Analysis of Functional NeuroImages) [32] and FSL (Oxford Centre for Functional Magnetic Resonance Imaging of the Brain, Oxford, UK). Fig 7 provides results from the DTI fractional anisotropy analysis for sagittal brain images. In this figure, DTI results from the standard (single-band) acquisition are compared to data acquired with the dual-band Fourier, SLR and wavelet refocusing pulses. The images acquired with the SLR and wavelet refocusing pulses achieved lower SAR compared to the images from the standard single band acquisition.

To provide an estimate of relative specific absorption rate (SAR), the integral of B1 squared values are listed in Table 1 along with the measured 10-second average SAR during human brain imaging, after initial SAR estimates were made on phantoms and *in silico*. While running the 2-band pulses on the 7.0T scanner, the pulse sequence included a separate fat-saturation pulse, a 2-band excitation pulse and a 2-band wavelet-optimized refocusing pulse. The real-time SAR monitor on the scanner displayed a 10-second average value of 1.1 W/Kg with the SLR pulse and 1.0 W/Kg with the wavelet-optimized pulse for the human scan while acquiring 18 slices within a 4.0 second TR. The RF transmit gain was held constant across the three scans acquired with the Fourier, SLR and wavelet pulses. In contrast, the standard (single-band) DTI pulse sequence on the 7.0T scanner generated a 10-second average SAR value of 1.6 W/Kg for 36 slices in a 4.0 second TR period. The SAR was significantly higher for the standard (single-band) scan, compared to the dual-band scans, because twice the number of slices needed to be acquired in the same TR period. Consistent with other work demonstrating multiband SAR reduction [33], multiband SMS imaging can be used as a method for SAR reduction.

## Discussion

This work demonstrates the application of wavelet domain optimization to RF pulse design. Wavelet domain optimization is an approach for designing ideal RF waveforms with a high degree of freedom. The cost function may be customized based on the desired application. Cost function terms, for example, may include peak B1 amplitude control of the pulse, regularization on the power deposition of the pulse, control of the slice profile magnitude and phase, the net vector sum of the transverse magnetization (M_x_-M_y_) across a slice pass band, and rejection band ripple control. By starting solely with wavelet decomposition on a Fourier pulse and no other prior knowledge of a pulse outcome, an optimized pulse can be designed with similar desirable slice profile characteristics of an SLR pulse with the added benefit of a small reduction in peak RF amplitude. If a greater reduction in peak B1 is desired, one could employ existing advanced SLR design techniques for peak B1 reduction [22,25,26,30] or wavelet optimization with unconstrained phase [34].

The dynamic wavelet reconstruction yields a high-resolution time domain pulse (3200 points with a 2us dwell time) intended to match the digital resolution of the pulse when loaded to the MRI scanner. One strength of the proposed wavelet optimization method is the ability to generate time domain pulses with a high temporal resolution and short dwell times on the order of a few microseconds or less. A very short dwell time is critical to design SMS pulses containing slice pass bands that are spectrally distant from the center carrier frequency. As the offset frequency of a slice pass band increases relative to the carrier frequency, the rate of amplitude modulation increases accordingly, requiring a shorter dwell time to digitally represent the amplitude oscillation of the waveform.

Although relaxation effects were not a significant factor for the relaxation times utilized in this optimization, this method has the expanded capability to include relaxation effects in the design optimization. Relaxation effects may be useful when designing a pulse to target tissue with ultra short T_1_ or T_2_ relaxation times, such as skeletal bone. This capability is a unique strength of the proposed wavelet pulse design method compared to existing methods that typically neglect relaxation effects for simplification.

A challenge of this demonstrated wavelet method is the computational time required to design wavelet-optimized pulses. For this reason, pulses need to be generated in advance of scanning and loaded to a database on the scanner, rather than generating the pulses in real-time on the scanner while prescribing the scan parameters. To improve computational speed, a single isochromat for Bloch Simulation was found to yield the same effective pulse outcome as 20 isochromats after iterative wavelet optimization. Computing in parallel in MATLAB with 4-cores on an Intel-i7-3770K processor, the optimization takes 70 minutes to reach convergence at 145 iterations involving 18659 Bloch Simulation function evaluations. It was observed that minimal change occurred in both the cost function value and the time domain waveform from iteration 115 through convergence at iteration 145. In practice, the computational speed could be improved by relaxing the stopping criteria if desired.

The phase across the slice pass bands was controlled through a term in the cost function that seeks to maximize the net vector sum of the transverse magnetization for each slice pass band. A slightly different approach would involve penalizing the variance in the phase across the slice pass band rather than maximizing the vector sum across the same pass band. We found in empirical trials that the vector sum is more effective than the approach of penalizing variance in the phase. In summary, the vector sum cost function term achieves the desired outcome of optimal slice profiles paired with phase coherence for maximum signal.

Alternatively, a different design approach for wavelet optimization is to leave the phase unconstrained and optimize purely on the magnitude of the spin magnetization. Such an approach significantly increases the potential impact of the wavelet optimization on lowering peak B1 amplitude [34]. Specifically, this approach is suitable for implementation where unconstrained phase across the pass band is acceptable, such as saturation, suppression, inversion recovery and twice-refocused spin echo sequences. Further, refocusing pulses with nonlinear phase across the slice are acceptable for three-dimensional spin echo imaging at 7.0T [35]. Excitation and refocusing pulse pairs designed with nonlinear phase [25,26] is another potential implementation when a single refocused spin echo experiment is desired.

Optionally, an additional penalty term can be added to minimize the integral of B1 squared, which is related to Specific Absorption Rate (SAR). Yet, in our simulations, minimizing the stop band ripple yielded a similar effect to an integral of B1 squared cost function term. If a constraint on the SAR limit for a given pulse were required, it would be effective to include a regularization term based on the integral of B1 squared value.

The wavelet-optimized pulse described in this work was designed as a purely amplitude-modulated pulse. However, this optimization method may be extended to design a pulse that is both amplitude and frequency modulated. This can be accomplished by preforming wavelet decomposition and reconstruction on the real and imaginary components separately of the RF pulse. Consequently, the real and imaginary components can be combined to generate a complex RF waveform to run through Bloch Simulation for cost function analysis. Additional design flexibility may be obtained by using wavelet optimization to design excitation and refocusing pulse pairs with non-linear phase. By designing the two pulses in pairs, the nonlinear phase across the pass band can be reversed by the refocusing pulse.

The spectrum of the Discrete Meyer Wavelets, after Fourier transform, yields two distinct bands, making this prototype wavelet especially suitable for multiband SMS pulse design. Although not addressed in this work, custom prototype wavelets may be designed to provide a higher level of refinement. A higher level of compression and improved conditioning of the minimization may be achieved by designing an optimal wavelet prototype for the desired target slice profile. For example, to optimize a pulse for a three-band slice profile, a custom prototype wavelet exhibiting three spectral bands could be designed to further improve compression. In addition to traditional rectangular slice profiles, the methods described in this work may be used to design RF pulses capable of generating arbitrary slice profiles.

## Conclusion

Existing pulse design methods are commonly based on analytic and discrete filter design algorithms. This work demonstrates a purely numerical method in the wavelet domain yielding an RF waveform that has been demonstrated to achieve a slice profile similar to an SLR pulse. Compared to the SLR pulse, the wavelet-optimized pulse achieves a 10.7% reduction in peak B1 and a 9.6% reduction in the integral of B1 squared, which is related to SAR. The wavelet optimization used a constant slice select gradient; hence, the pulses are not dependent on gradient slew rate. Moreover, wavelet domain optimization allows design of digital waveforms with very short dwell times while attaining a high degree of design freedom. Arbitrary and unique slice profiles may be chosen and the terms of the cost function may be further customized to achieve a desired outcome.
